# Statistics design for the synthesis optimization of lignin-sulfonate sulfur-doped mesoporous carbon materials: promising candidates as adsorbents and supercapacitors materials

**DOI:** 10.1038/s41598-024-75003-1

**Published:** 2024-10-07

**Authors:** Glaydson Simoes dos Reis, Shaikshavali Petnikota, Helinando Pequeno de Oliveira, Irineu A.S. de Brum, Mikael Thyrel, Guiherme Luiz Dotto, Eder Claudio Lima, Mu. Naushad, Tao Hu, Ulla Lassi, Alejandro Grimm

**Affiliations:** 1 Department of Forest Biomaterials and Technology, Biomass Technology Centre, Swedish University of Agricultural Sciences, Umeå, SE-901 83 Sweden; 2 Laboratory of Industrial Chemistry and Reaction Engineering, Faculty of Science and Engineering, Åbo Akademi University, 20500 Åbo/Turku, Finland Finland,; 3 Institute of Materials Science, Federal University of Sao Francisco Valley, Juazeiro, 48902-300 BA Brazil; 4 Mineral Processing Laboratory, Federal University of Rio Grande do Sul, 9500 Bento Gonçalves Avenue, Porto Alegre, 91501-970 Brazil; 5 Research Group on Adsorptive and Catalytic Process Engineering (ENGEPAC), Federal University of Santa Maria, Av. Roraima, 1000-7, Santa Maria, 97105-900 RS Brazil; 6 Institute of Chemistry, Federal University of Rio Grand do Sul (UFRGS), Porto Alegre, RS Brazil; 7 Department of Chemistry, College of Science, King Saud University, P.O. Box 2455, Riyadh, 11451 Saudi Arabia; 8 Research Unit of Sustainable Chemistry, University of Oulu, P.O. Box 3000, Oulu, FI-90014 Finland

**Keywords:** Lignin sulfonate, Sulfur doping, Sulfur-doped carbons, Adsorption, Sodium diclofenac, Supercapacitors, Orange 16 dye, Materials science, Green chemistry

## Abstract

This study employed lignin-sulfonated (LS) to develop biobased carbon materials (LS-Cs) through a sulfur-doping approach to enhance their physicochemical properties, adsorption capabilities, and energy storage potentials. Various characterization techniques, including BET surface area analysis, SEM imaging, XPS, Raman spectroscopy, and elemental composition (CHNS), were employed to assess the quality of the LS-Cs adsorbent and electrode samples. Response Surface Methodology (RSM) was utilized for optimizing the two main properties (specific surface area, A_BET,_ and mesopore area, A_MESO_) by evaluating three independent factors (i.e., activation temperature, ZnCl_2_:LS ratio, and sulfur content). According to the statistical analysis, A_BET_ and A_MESO_ were affected by ZnCl_2_ and sulfur content, while the pyrolysis temperature did not affect the responses in the studied conditions. It was found that increasing the ZnCl_2_ and sulfur contents led to an increment of the A_BET_ and A_MESO_ values. The LS-C materials exhibited very high A_BET_values up to 1993 m^2^ g^−1^ and with predominantly mesoporous features. The S-doping resulted in LS-Cs with high sulfur contents in their microstructures up to 15% (wt%). The LS-C materials were tested as adsorbents for sodium diclofenac (DCF) adsorption and reactive orange 16 dye (RO-16) and as electrodes for supercapacitors. The LS-Cs exhibited excellent adsorption capacity values for both molecules (197–372 mg g^−1^) for DCF, and (223–466 mg g^−1^) for RO-16. When tested as electrodes for supercapacitors, notably, LS-C3, which is a doped sample with sulfur, exhibited the best electrochemical performance, e.g. high specific capacitance (156 F/g at 50 mV/s), and delivered an excellent capacitance after 1000 cycles (63 F/g at 1 A/g), which denotes the noteworthy capacitive behavior of the S-doped electrode. Thus, the present work suggests an eco-friendly resource for developing effective, productive carbon materials for adsorbent and electrodes for SC application. However, further studies on the complete application of these materials as adsorbents and electrodes are needed for a deeper understanding of their behavior in environmental and energy storage applications.

## Introduction

Biobased industries’ conversion of biomass residues into carbon-based materials represents an opportunity to create value from by-products, reduce reliance on fossil resources, and reduce costs associated with production processes^1^. Biomass-derived carbon materials (BioCMs) can be obtained from biomass-based resources prepared through different thermochemical processes^2,3^. BioCMs have distinct properties such as high surface area, pore networks with various geometries, and surface functionalities, properties that make them highly suitable for a wide range of applications such as adsorbents to remove pollutants from wastewater^3–5^and as electrode materials for supercapacitors^6–8^. The final characteristics of BioCMs are commonly a function of the type of biomass employed as a precursor, as well as different thermochemical treatments used for their production^9^. However, the state-of-the-art presents gaps between methods to produce different carbon materials from biomasses and how their properties are connected to the resulting carbon performances for use as adsorbents or as electrodes in energy storage devices^10^.

Recent research has focused on heteroatom-doping, from creating and improving new functionalities of these carbon-doped materials to broadening the spectrum of their applications^11,12^. Heteroatom-doping represents an efficient strategy to create a promising class of suitable materials for environmental and energy applications^11,12^. Unlike pristine carbon materials, which are predominantly composed of carbon atoms, heteroatom-doped BioCMs have incorporated heteroatoms such as nitrogen, oxygen, sulfur, phosphorus, and boron into their carbon matrix^13^, which introduces unique chemical functionalities and surface properties, enabling tailored interactions with target molecules or improving electrochemical properties^3,5,13^. For instance, due to their improved conductivity, sulfur-doped activated carbons have remarkably performed as adsorbent and electrode materials. The versatility of heteroatom doping opens avenues for developing multifunctional materials with tailored properties to suit specific applications from a wide range of biomass-based precursors, including from the pulp and paper industry^14^.

The pulp and paper industry is a cornerstone of modern society, playing a pivotal role in various sectors ranging from packaging and publishing to textiles and construction^15–17^. At the heart of the pulp and paper industry is converting raw materials, primarily wood fibers, into a wide array of paper and paperboard products. This process involves several stages, including pulping, bleaching, refining, and papermaking, each contributing to the final product’s properties^17^. Alongside producing paper and pulp, the industry generates substantial waste biomass^15,17^. It is estimated that for every ton of paper produced, the industry generates approximately 0.5 to 1.0 tons of waste biomass, which may constitute spent pulping liquors, sludge, and wood residues, including lignin-based wastes such as lignin-sulfonate [18], which make mandatory finding ways to recycling and recovery these wastes into new materials/products^19^.

The lignin sulfonate is a product from cellulose extraction through a sulfite pulping process that involves a liquor rich in sulfur dioxide (SO_2_) for cooking the wood resources. The lignin dissolved in the pulping liquor undergoes sulfonation, a chemical reaction in which sulfonic acid groups (-SO_3_H) are introduced onto the lignin molecule^20^. Typical applications of lignin sulfonates are plasticizers in concrete, binders in particle boards, flooring, and coal briquettes, a constituent of the coating material used in lead-acid battery grids, dust suppression agent for dirt roads, additive in feeds, ensilage, and flame retardants, among others^18^.

This study utilized lignin sulfonate sourced from a Swedish Kraft paper industry as a biomass feedstock for producing BioCMs, intended for use as adsorbents and in supercapacitor electrodes. Chemical activation was achieved using ZnCl_2_, and heteroatom doping was performed with elemental sulfur to tailor the physicochemical properties of lignin sulfonate-based BioCMs. Experiments were conducted using a design of experiments (DoE) approach to optimize preparation conditions, aiming for BioCMs with increased specific surface areas (S_BET_) and high mesopore areas (A_MESO_). With a similar electronegativity to carbon (2.55), sulfur (2.58) was chosen as a dopant to narrow the energy gap between molecular orbitals and foster thiophene group formation on the surface of the BioMCs, which are beneficial to bind molecules and store ions. Thus, this research endeavors to bridge research gaps and offer insights into the properties of BioCMs concerning the influence of pyrolysis/activation and doping processes.

## Materials and methods

### Materials

Lignin sulfonate (LS) samples utilized in this investigation were sourced from a Kraft paper mill in Sundsvall, Sweden. Elemental sulfur and zinc chloride of analytical grade were purchased from Sigma Aldrich.

### BioCMs preparation

The lignin sulfonate-based BioCMs were synthesized via a one-step pyrolysis activation process following a previously documented protocol. Initially, LS (15 g) was mixed with ZnCl_2_, and approximately 30.0 mL of water was added during blending to ensure uniform paste formation. Subsequently, the paste underwent drying in an oven set at 105 °C for 24 h, followed by pyrolysis in a reactor externally heated by an electric oven. The pyrolysis process was conducted at a constant heating rate of 10 °C/min under anoxic conditions. Upon reaching the designated temperature, the sample was held for 1 h. The reactor was allowed to cool to room temperature in the post-treatment step. To eliminate residual ZnCl_2_ and ashes, the sample underwent a reflux process for 2 h at 75 °C with a 1.0 M HCl solution. Subsequently, it was washed with distilled water until the washing fluid achieved a neutral pH.

### Experimental design

A Box-Behnken design (BBD) was employed to conduct the LS pyrolysis and activation process. BBD is an incomplete block design consisting of a minimum of three factors. Each block consists of four combinations of these three factors, one always positioned at the central point while the others vary between the lower (-1) and upper (+ 1) limits. The pyrolysis temperature (°C), zinc chloride/LS dry matter ratio (-), and sulfur/LS dry matter ratio (-) were chosen as design variable factors. The design comprised 15 experiments with three center points and 12 factorial points (see Table 1), with the upper and lower limits determined based on literature references.

The responses selected for analysis included the B.E.T. (Brunauer-Emmett-Teller) surface area (S_BET_, m^2^/g), micropore surface area (S_MICRO_, m^2^/g), mesopore surface area (S_MESO_, m^2^/g), and pore volume (cm^3^/g) (Table 1). The impact of the design factors on the responses and determining optimal values were evaluated using the Minitab software.

**Table 1:** Box-Behnken design (BBD) experimental design.

StdOrder	RunOrder	PtType	Temperature (°C)	Zinc Ratio (-)	Sulfur content (%)
1	13	2	600	1	25
2	2	2	800	1	25
3	3	2	600	3	25
4	4	2	800	3	25
5	5	2	600	2	0
6	6	2	800	2	0
7	7	2	600	2	50
8	8	2	800	2	50
9	9	2	700	1	0
10	10	2	700	3	0
11	11	2	700	1	50
12	12	2	700	3	50
13	1	0	700	2	25
14	14	0	700	2	25
15	15	0	700	2	25

### Characterization

Surface area characterization was done using data from nitrogen isotherms following standard procedures. Specific surface area (SSA) values were derived using the BET method, while pore size distribution curves were obtained using the BJH principle. The contents of carbon (C), oxygen (O), hydrogen (H), and sulfur (S) of the produced carbons were conducted using an elemental analyzer (EA-IsoLink, Thermo Fisher Scientific) following standard procedures. Raman spectra were acquired using a Bruker Bravo spectrometer (Bruker, Ettlingen, Germany) in the spectral range between 800 and 2000 cm^−1^ with 254 scans at 4 cm^−1^ resolution. The carbon materials’ hydrophobic-hydrophilic index (HI) was determined according to the methodology described in Ref. [5]. X-ray photoelectron spectroscopy (XPS) analysis was performed using a Thermo Fisher Scientific ESCALAB 250Xi XPS System, adhering to standard measurement procedures.

### Preliminary adsorption tests

To assess the adsorption capacity of the BioCMs for a specific water pollutant, all produced BioCMs were utilized to remove sodium diclofenac (DCF) from the aqueous solution. BioCM (30 mg) was contacted with 20 mL of DCF (C_o_=700 mg/L). The samples were then agitated using a shaker at 300 rpm for 3 h. Subsequently, the samples were centrifuged to effectively separate the solid and liquid phases. The liquid phase was retrieved to determine the remaining amount of DCF post-adsorption, utilizing a Shimadzu UV-visible spectrophotometer (model 1800) at λ_max_ of 276 nm. The adsorption capacity of the CMs was calculated using Eq. (1), where C_o_ and C_f_ are the initial and final DCF concentrations, respectively, m is the mass of adsorbent (g), and V is the DCF volume (L).

$$\:q=\:\frac{({C}_{0}-{C}_{f})}{m}.V$$

### Preliminary supercapacitor tests

Electrode slurries were prepared by mixing S-doped activated carbons (S-ACs), PVDF binder, and Super P Carbon in NMP solvent in 80:10:10 wt%, respectively. The prepared electrode inks were dropped cast on 15 mm circular discs of FuelCell AvCarb MGL370 carbon paper. The coated electrodes were dried at 80 ℃ overnight. The active mass loaded onto the electrodes was in the range of 4–7 mg. 

Coin cell (CR2032) symmetric supercapacitors (SCs) of the fabricated electrodes with similar mass loading were assembled inside an argon-filled glovebox (MBraun). As an electrolyte, a mixture of 1-Butyl-3-methylimidazolium tetrafluoroborate ([BMIM]BF4, Sigma Aldrich) and Acetonitrile (Sigma Aldrich) in 1:1 wt% was used. Whatman glass microfiber filter paper was used as a separator. 

The electrochemical measurements of the assembled coin cell SCs were carried out after relaxing overnight. Cyclic voltammetry (CV) was conducted at scan rates ranging from 200 to 10 mV/s in the voltage window of 0.0–2.5 V using BioLogic SP-150e potentiostat. Galvanostatic cycling (charge-discharge) measurements were carried out at a 1.0 A/g current rate in the voltage window, the same as the CV, using a BioLogic BSC-800 series battery cycler.

## Results and discussion

### Porosity data

The results from the characterization of the produced ACs are given in Table 2. Regarding textural properties, all produced activated carbons (ACs) highlighted high specific surface areas (S_BET_), regardless of the experimental conditions. The highest S_BET_value of 1993 m^2^g^[-1^ was achieved at 600 °C with a ZnCl_2_ ratio 3 (LS-C3). This configuration, characterized by a lower pyrolysis temperature, a higher ZnCl_2_ ratio, and a moderate sulfur (S) ratio, yielded the optimal outcome. Additionally, across other experimental setups, ACs with S_BET_values exceeding 1700 m^2^g^[-1^ were generated (Table 2), indicating the efficacy of lignin sulfonate as a precursor for producing ACs with significant specific surface areas and well-developed porosities. 

Analysis of S_MESO_ and S_MESO%_ values revealed that the lignin sulfonate-derived ACs predominantly exhibited mesoporous structures. For instance, the relative mesopore distribution was 100% (LS-C12), 94,2% (LS-C4), 93,9% (LS-C3), and 93,7% (LS-C7). The presence of mesopores is particularly advantageous for adsorption applications as they facilitate wetting and transporting the liquid containing contaminants throughout the bulk of the AC. On the other hand, LS-C9 (produced at 700 °C with a ZnCl_2_ ratio of 1) and LS-C13 (produced at 600 °C with a ZnCl_2_ ratio of 1 and a sulfur ratio of 0.25) were dominated by microporous structures, representing 74.0% and 62.1% of the total S_BET_, respectively.

**Table 2:** Textural properties and yield values of the ACs.

Samples (Temp.:Zn: S)	Coded samples	S_BET_ (m^2^ g^−1^)	S_MESO_ (m^2^ g^−1^)	S_MICR_ (m^2^ g^−1^)	S_MESO%_ (%)	S_MICR%_ (%)	Pore volume (cm^3^ g^−1^)	Average pore diameter (nm)
700:2:0.25	LS-C1	1076	796	280	74.0	26.0	1.00	3.72
800:1:0.25	LS-C2	498	195	303	39.1	60.8	0.61	4.91
600:3:0.25	LS-C3	1993	1871	122	93.9	6.10	2.21	4.73
800:3:0.25	LS-C4	1621	1527	94	94.2	5.80	1.58	3.89
600:2:0	LS-C5	911	380	531	41.7	58.3	0.64	2.80
800:2:0	LS-C6	822	415	407	50.5	49.5	0.75	3.67
600:2:0.5	LS-C7	1088	1019	69	93.7	6.30	1.31	4.80
800:2:0.5	LS-C8	1213	1002	211	82.6	17.4	1.23	4.06
700:1:0	LS-C9	262	68	194	26.0	74.0	0.32	4.87
700:3:0	LS-C10	753	588	165	78.1	21.9	0.99	5.29
700:1:0.5	LS-C11	598	279	319	46.7	53.3	0.62	4.13
700:3:0.5	LS-C12	1758	1758	0	100	0.00	1.62	3.69
600:1:0.25	LS-C13	475	180	295	37.9	62.1	0.51	4.33
700:2:0.25	LS-C14	807	599	208	74.2	25.8	0.86	4.25
700:2:0.25	LS-C15	828	638	190	77.1	22.9	0.87	4.19

### Response surface plots, influence of the factors, and statistical analysis

**Fig. 1:**
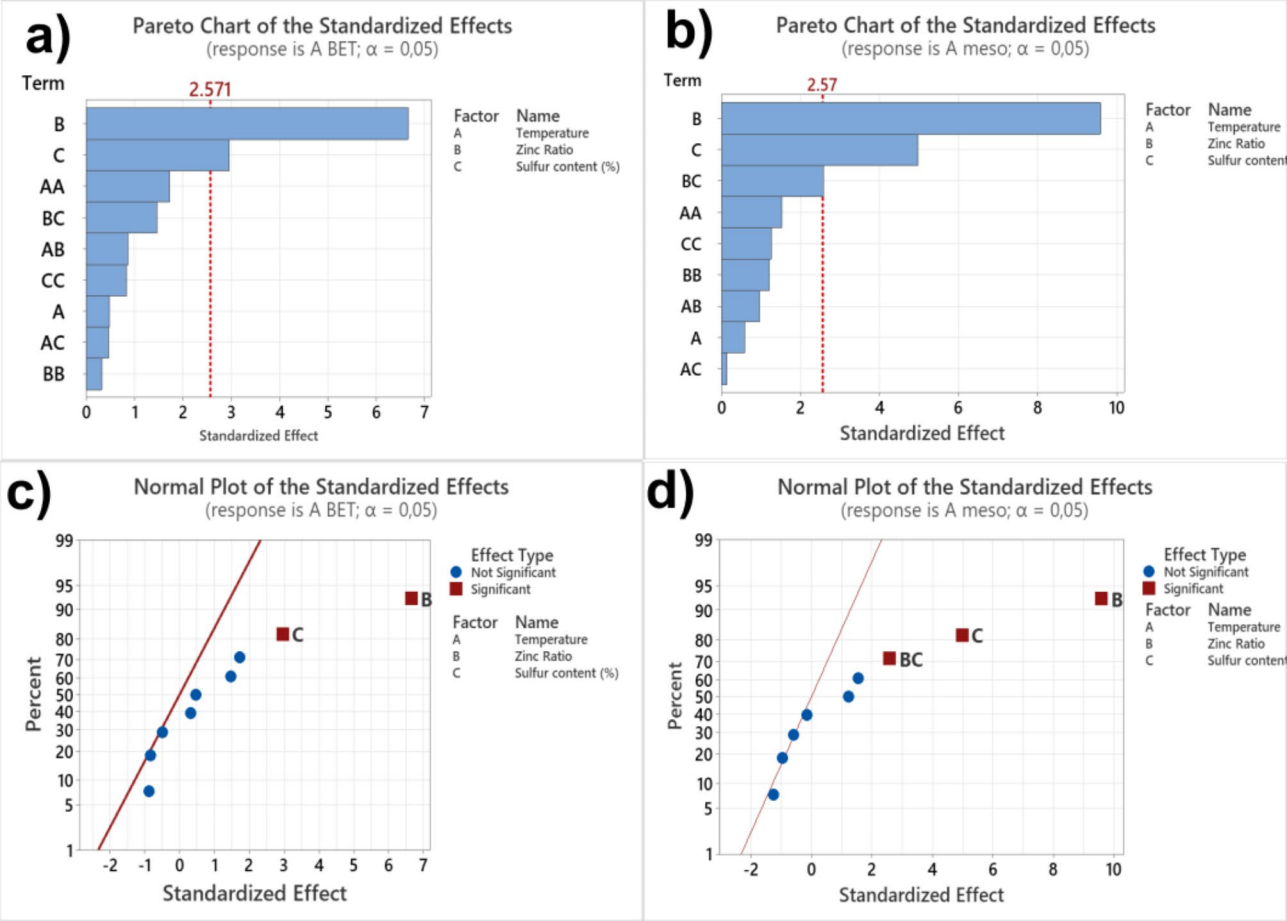
Pareto charts of the (a A_BET_ and b, A_MESO_) standardized effects of chosen parameters (pyrolysis temperature, the ratio of ZnCl_2_, and sulfur content on A_SBET_ and A_MESO_. Typical plots of the (c, A_BET,_ and d, A_MESO_) standardized effects of chosen parameters (pyrolysis temperature, ratio of ZnCl_2_, and sulfur content on A_SBET_ and A_MESO_.

Based on the design experimental data, the factors’ effect and their interactions on the chosen variables S_BET_ and S_MESO_ are statistically examined through the Pareto chart, which graphically demonstrates what the significant factor (s) and their interaction (s) on the chosen variables (S_BET_ and S_MESO_)^4,21^. The Pareto chart exhibits bars that visualize the factors and their interactions^4,21^. The dotted line is associated with the p-value corresponding to each studied factor and their interactions; the bar touching this line means it significantly influences the chosen variables^4,21^. For the S_BET_ response, this chart displays that the ratio of ZnCl_2_ and sulfur content were the variables affecting the S_BET_ values (see Fig. 1a), while the range of the studied temperature did not affect the S_BET_ values. For the S_MESO_ values, the ratio of ZnCl_2_ and sulfur content were the variables that most influence factors affecting the S_BET_ values (see Fig. 1b), followed by the interaction of the same (ratio of ZnCl_2_ and sulfur content). This means that only ZnCl_2_and sulfur content were statistically significant under the range of the studied factors, while all the others were not^4,14,21^.

The Pareto chart is a helpful tool for quickly identifying the most influential factors affecting the chosen responses (e.g., S_BET_ and S_MESO_). However, by this chart, it is impossible to indicate and understand how this influence occurred (positively or negatively). In this sense, the Normal plot gives such information (see Fig. 1c, d). The typical standard plot shows the magnitude, direction, and importance of the effects on the studied process over the chosen responses. Moreover, it highlights that a positive impact enhances the reaction (e.g., S_BET_ and S_MESO_) when the variable conditions change from low to high value, while a negative implies in a decrease the response when the variable conditions change from low to high value.

Considering this information, the normal plot in Fig. 1c, d suggest that the ZnCl_2_ and sulfur content ratio positively influenced both S_BET_ and S_MESO_ values. Moreover, the interactions between ZnCl_2_ and sulfur content (BC) also positively impacted S_MESO_ value. This means that enhancing the amount of ZnCl_2_ and sulfur increases the specific surface area value and the number of mesopores in the carbon material structures.

**Fig. 2:**
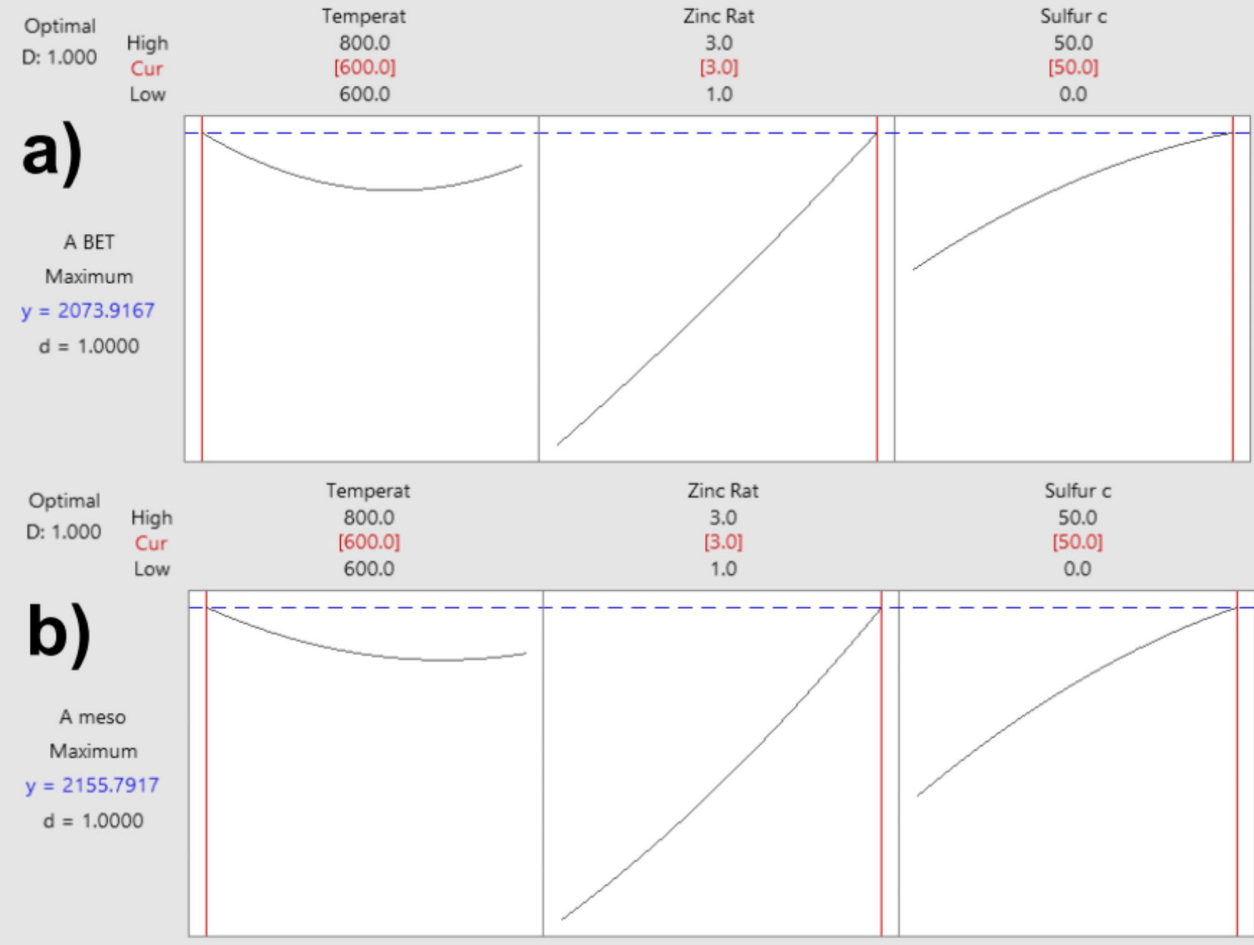
Composite desirability with the two responses, A_BET_ and A_MESO_.

Optimization is calculated by combined desirability ranges from 0 to 1^21^. For the optimization experiment, the desirability (D) and individual desirability (d) values indicate that the process of LS-C preparation with the desired properties was well optimized since these values reached 1.0, which is an excellent score, which means that both responses (A_BET_ and A_MESO_) were very close in their absolute settings^21^. Therefore, based on the outcomes above, it is possible to state that, within the outlined conditions in this work, the optimization of the S-doped LS-C carbon materials properties (A_BET_ and A_MESO_) should be two parameters (Temperature and S-content). Figure 2 presents the composite desirability measures, a graph that measures the overall predictability for both responses (A_BET_ and A_MESO_) by the predictor parameters^14^. Interestingly, the overall desirability for optimizing the maximum A_BET_ and A_MESO_ was the same: 800 °C, 3 h, and 50% sulfur.

### Raman spectroscopy analysis of the lignin-sulfonate doped carbons

**Fig. 3:**
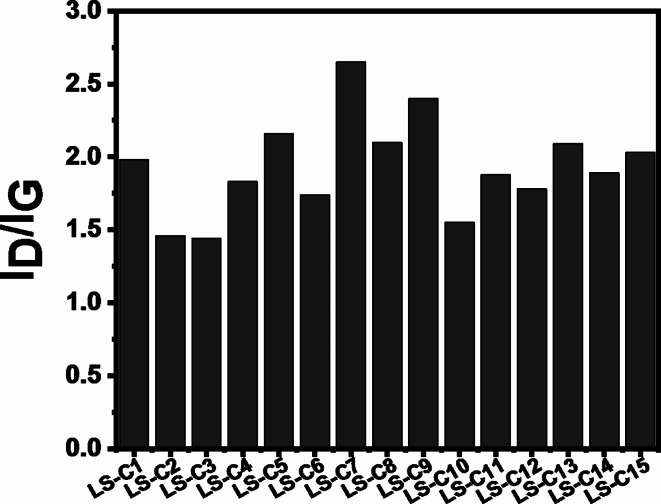
Raman analysis. The ratio of I_D_/I_G_ bands of the LS-C materials.

The evaluation of the graphitization/defect degree (through Raman spectroscopy analysis) of a carbonaceous material is an important aspect to be studied, considering that crystalline graphitic structure or level of defects may impose noteworthy properties/performances of the carbon materials related to the ending application (adsorption of pollutants from wastewaters or electrodes for supercapacitors). Raman spectroscopy of biomass-based carbon materials provides two important main peaks/bands at around 1500–1650 cm^−1^(G band) that are related to in-plane stretching vibration of the sp^2^ hybridized carbon atoms (planar carbon structure) and a second band around 1300 cm^−1^(D band) that is attributed to the sp^3^C atoms (tetrahedral carbon structure), whose its presence in the LS-C materials suggest defects in carbon framework^22,23^. A ratio I_D_/I_G_can be obtained from these peaks, indicating the degree of graphitisation of the LS-Cs^24–26^. Figure 3 shows that I_D_/I_G_values are all higher than 1, highlighting the larger defectuous structure in the LS-C materials instead of the dominant graphitic/crystalline structure^3,27^. This result shows that all LS-C materials have predominant defects despite the experimental preparation conditions, impregnation with ZnCl_2_, and S-doping. It is reported that carbon-based materials rich in defects can be effectively employed as adsorbents and electrodes because defects can act as active adsorption sites that help to boost the material’s adsorptive properties and electrolyte penetration into the carbon-defective structure^24,28,29^.

### Elemental composition and XPS analysis of the LS-C materials

The samples were subjected to the elementary analysis (CNHS) method to evaluate sulfur incorporation into the LS-carbon structures successfully. Table 3 displays the quantitative composition of the main elements such as carbon (C), Nitrogen (N), Hydrogen (H), Oxygen (O), and Sulfur (S). As a comparison, a commercial AC has 88% of C, 0.5% of H, 0.5% of N, and 3–4% ash^30^. The LS-C samples exhibited high C contents varying from 74.66 to 85.62 wt%, lower than the commercial carbon; however, the lower carbon content is related to the higher sulfur content in the original lignin sulfonate. Regarding the sulfur content, the samples not doped exhibited the lowest sulfur content (LS-C5, LS-C6, LS-C9, and LS-C10, See Table 3). The LS-C samples in which sulfur was incorporated exhibited elevated S contents, proving the successful doping process. Elevated S content into the carbon matrix can yield benefits when they are employed as adsorbents for adsorbing pollutants from waters and enhancing the LS-Cs’ electrochemical performance for application as electrodes in supercapacitors.

Zhang et al^31^. doped a graphene material with sulfur atoms. They highlighted that such a strategy introduced many defective sites, which generated changes in the electron distribution of the carbon material, and these changes are beneficial to binding molecules and storing ions. Moreover, the S doping created more active regions on the carbon surface, such as at the edges and the neighbour sites of S atoms, which would facilitate attraction/bind molecules. Incorporating sulfur atoms into the carbon matrix can create abundant active sites beneficial to adsorptive and electrochemical reactions^32,33^.

**Table 3:** Elemental composition of the LS-C materials.

Samples	Carbon (%)	Nitrogen (%)	Hydrogen (%)	Oxygen (%)	Sulfur (%)	Ash (%)
LS-C1	81.43	1.56	1.13	4.0	6.40	5.48
LS-C2	77.55	1.33	0.78	4.7	7.46	8.18
LS-C3	77.33	1.54	1.26	4.4	12.08	3.39
LS-C4	82.45	1.05	1.11	4.2	5.22	5.97
LS-C5	83.85	1.12	1.74	4.8	3.75	4.74
LS-C6	85.62	1.13	0.82	3.2	4.20	5.03
LS-C7	75.18	1.39	1.07	5.9	15.54	0.92
LS-C8	78.19	1.53	0.62	3.4	8.69	7.57
LS-C9	76.99	1.07	1.21	5.4	3.97	11.36
LS-C10	79.55	0.93	1.03	3.6	4.15	10.74
LS-C11	75.05	1.44	1.01	5.1	8.77	8.63
LS-C12	74.66	1.32	1.08	4.5	10.12	8.32
LS-C13	79.95	1.19	1.85	5.3	4.49	7.22
LS-C14	78.97	1.49	0.95	5.0	9.64	3.95
LS-C15	75.24	1.44	0.92	6.6	11.28	4.52

**Fig. 4:**
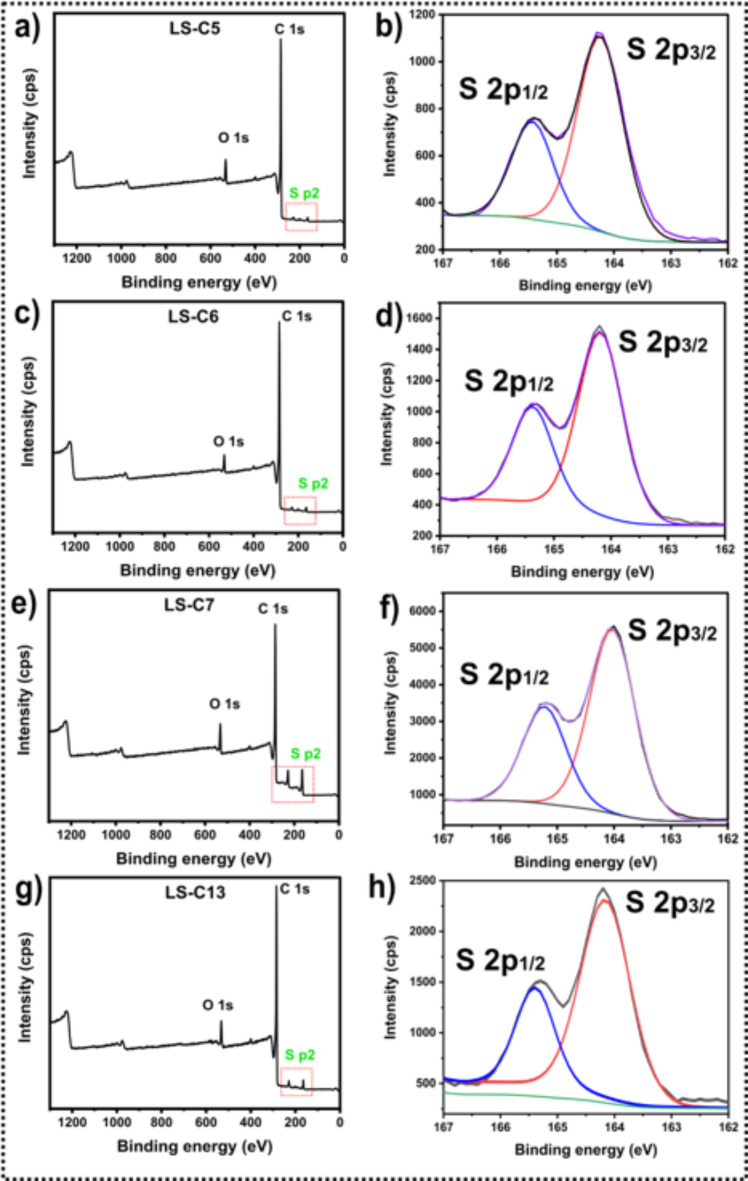
X-ray photoelectron spectroscopy. Survey spectra for (a) LS-C5, c) LS-C6, e) LS-C7 and g) LS-C13. High resolution S 2p spectra for (b) LS-C5, d) LS-C6, f) LS-C7 and h) LS-C13.

In carbon-based materials, the elemental chemical surface composition influences several functional groups, largely affecting the material’s adsorptive and electrochemical properties^24,27^. XPS further examined the surface chemical composition of the LS-C samples to assess better the influence of sulfur doping in their surface compositions/functionalities. By the survey spectra (Fig. 4a, c,e, g), four peaks can be observed, two near 284.1 and 532.1 and eV, corresponding to the peaks of C1s and O1s, respectively, and two peaks at 165.5 and 229.4 eV, which correspond to Sp2 peaks^24,27^. It is worth highlighting that the sulfur peaks are much higher in the doped samples due to their higher presence than the non-doped samples (LS-C 5 and LS-C6), indicating the successful introduction of S-atoms into the carbon matrix structure^24,27^. 

To further evaluate the nature state of the S element, the S peak was deconvoluted in their high-resolution spectra, which are shown in Fig. 4b, d,f, h. The S 2p spectra, in all samples, can be deconvoluted into two peaks located at around 164.1 eV and 165.3 eV, which is characteristic of S 2p_3/2_ and S 2p_1/2_of the C-S covalent bond in thiophene-S and sulfoxide, respectively^24,27,34^. The abundance of the sulfur states on the carbon surface suggests a high presence of functional groups that can boost both the adsorptive and electrochemical performances of the LS-C samples, which indicates that S-doping is a strategy to improve the performance of the carbon materials.

## TEM analysis of the LS-C samples

**Fig. 5:**
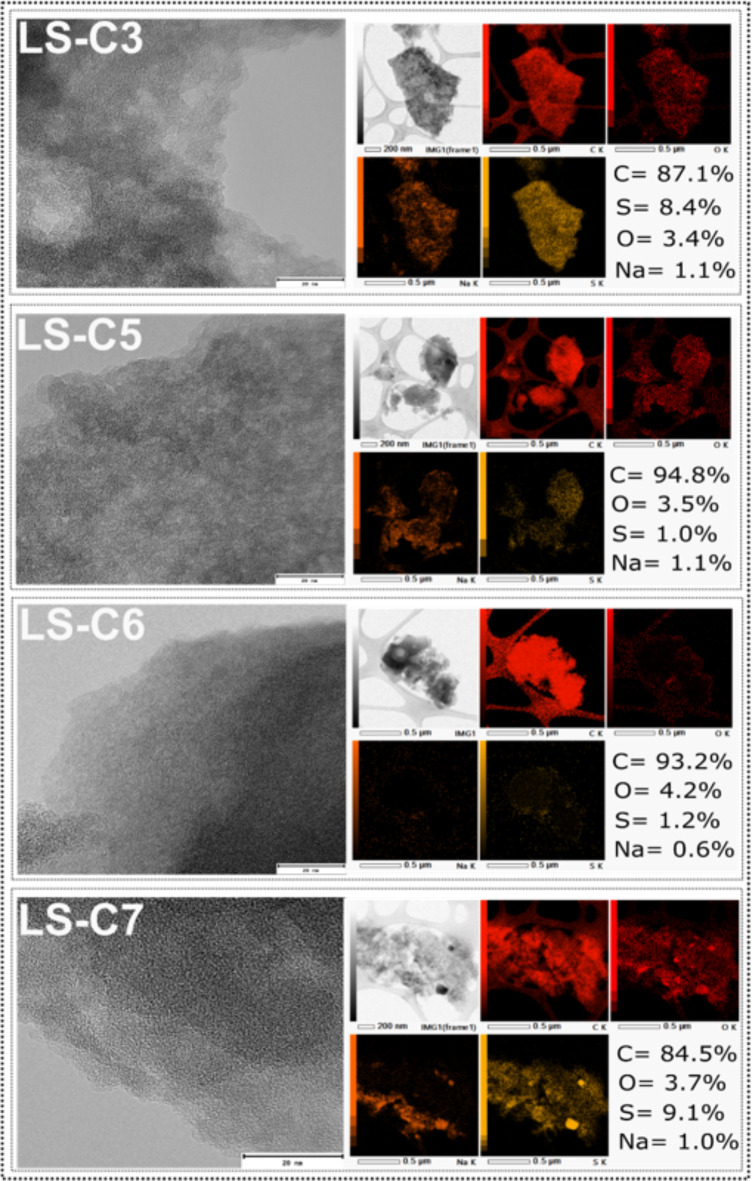
HRTEM images of the LS-C samples (scale bar represents 20 nm), and their corresponding STEM-EDX maps (scale bar represents 0.5 μm).

The microstructure of the LS-C materials was further evaluated by HRTEM (Fig. 5), which confirms the porous structural feature of the carbon materials. Despite the doping process, it is observed for all samples, mostly disordered/random (amorphous) structures and rich in defects. Defects in carbon materials could work as active sites for binding molecules and improving the interaction with ion or electrolyte adsorption and intercalation. Thus, based on TEM analysis, the doping and the pyrolysis conditions did not have a huge impact on the carbon arrangement of the LS-C samples since the displayed samples presented similar nanostructures. To further analyze the effect of doping on the LS-C materials, the elemental distribution within the samples was verified using STEM-EDX (Fig. 5).

It is observed that the samples without doping displayed very low sulfur content, which is present in the lignin-sulfonate sample, while the doped samples exhibited higher S-content. Besides, the STEM-EDX mapping results show that the sulfur is homogeneously distributed on the surface of the samples, indicating the successful S-doping of the LS materials. The homogeneous distribution of sulfur atoms over the LS-C surface could improve their adsorptive/electrochemical properties due to the more homogeneous distribution of sulfur active sites over the adsorbent/electrode surface, improving the contact solid/liquid reactions.

## Results on preliminary adsorption tests

**Fig. 6:**
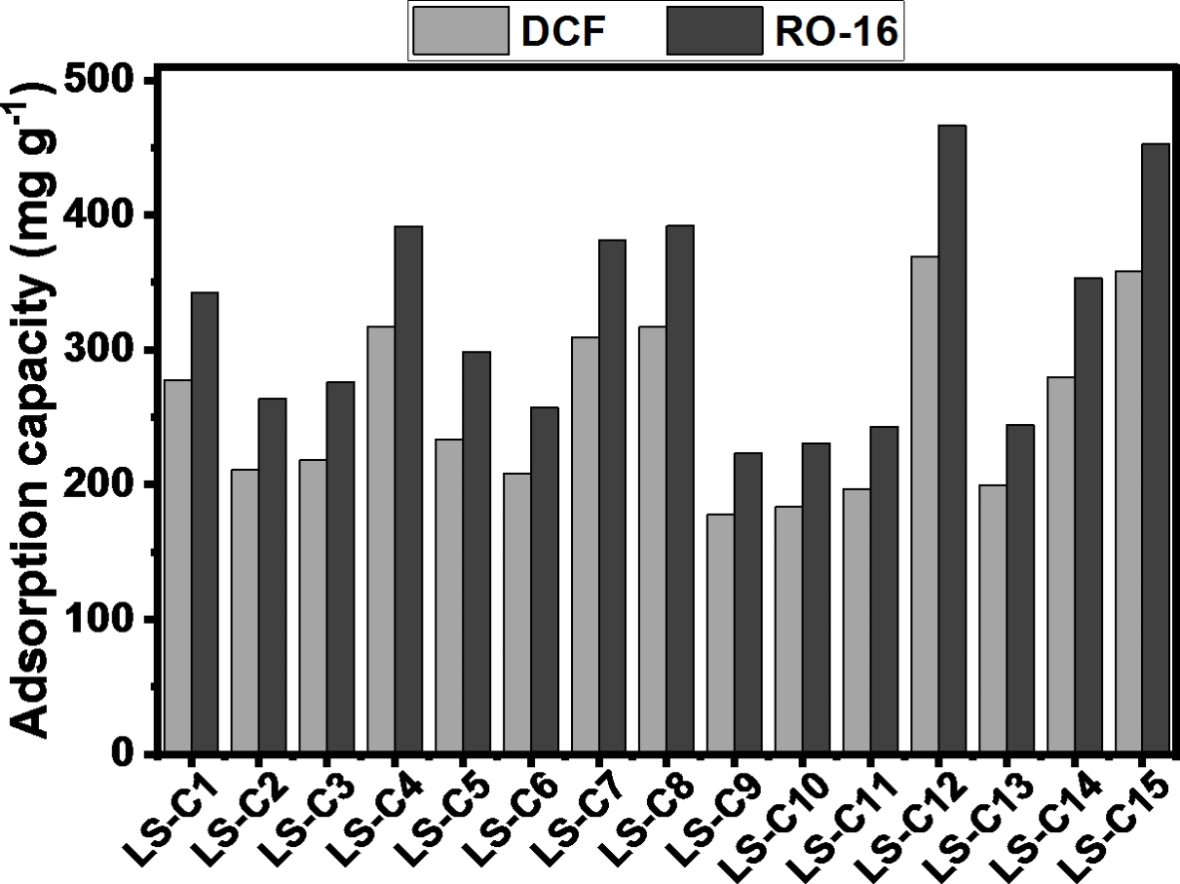
DFC and RO-16 adsorption capacity on LS-C materials.

Aiming to evaluate the suitability of the LS-C materials as potential adsorbents, they were employed in the removal of a drug (diclofenac sodium) and a dye (reactive orange 16) from aqueous solutions (Fig. 6). The LS-Cs exhibited excellent adsorption capacity values for both molecules (197–372 mg g^[-1^) for DCF and (223–466 mg g^[-1^) for RO-16. Such high adsorption capacities of all carbons for both dye and drug are explained due to the high surface area of the adsorbents. The LS-C materials possess well-developed pore structures containing both micro- and mesoporous that are highly effective in adsorbing small organic molecules such as DFC and RO-16. For instance, DCF and RO-16 have molecule sizes of 1.01 nm and 1.68 nm, respectively, which they can easily be accommodated in pores bigger than their size, which is the case of big micropores (up to 2 nm) and mesopores (from 2 to 50 nm). In addition, the materials have high surface functionalities, which may have also contributed to the high overall adsorption capacities. It is worthwhile to mention that the sulfur seems to have impacted the ability of the LS-C adsorbents to adsorb both DCF and RO-16 since the five most efficient adsorbents that presented the highest adsorption capacities were the ones doped with sulfur. However, even the samples non-doped with sulfur also exhibited very high adsorption capacities, as shown in the comparative Table 4, with other adsorbents found in the literature. The highest adsorption capacity (q) among all 15 LS-Cs was achieved by LS-C12, which. It was used with the different adsorbents, as reported in Table 4.

By comparison with the literature data, it is possible to observe that the adsorption capacity (q) of the best-performing AC (AC14) is even higher than the adsorption capacities of many different adsorbents reported in the literature (see Table 4). Compared to the other research, our LS-Cs seem much more effective since they present an easy and less complex preparation method compared to other less effective adsorbents shown in Table 4. For instance, the PVA/SA/CNC)@PEI adsorbent achieved a higher adsorption capacity for DCF (444.44 mg g^−1^) compared to LS-C12 (372 mg g^−1^). However, its preparation method is highly complex, and expensive chemicals and reagents are needed, making its actual use unsustainable and unrealistic. However, LS-C12 offers a more accessible synthesis with a sustainable resource (lignin-sulfonate), which leads to a lower production cost, classifying it as an excellent adsorbent (s) to remove DFC and RO-16 from aqueous solutions.

**Table 4:** Comparison of the adsorption capacities for diclofenac using different adsorbents.

Adsorbents	q (mg g^1^ )	Molecule	Ref.
Biochar from birch tree wastes	355	DCF	2
Selenium-doped biochar from birch tree wastes	434	DCF	2
Sewage sludge –polysiloxanes Composite	26.12	DCF	35
Reduced graphene oxide	59.67	DCF	36
Commercial AC	83	DCF	37
carbon nanotubes/alumina hybrid	33.86	DCF	38
Graphene oxide nanosheets	128.75	DCF	39
Carbon xerogel	80.0	DCF	40
PVA/SA/CNC)@PEI	444.44	DCF	41
Norway spruce bark AC (AC14)	417.4	DCF	2
S-doped lignin sulfonate carbons (LS-C12)	372	DCF	This study
Ulva Prolifera AC	232.0	RO-16	42
Psyllium seed powder	206.6	RO-16	43
Waste sawdust AC	58.54	RO-16	44
Groundnut shell AC	11.05	RO-16	^45^
Biochar from birch tree wastes	332	RO-16	2
Selenium-doped biochar from birch tree wastes	538	RO-16	2
Nanocomposite based on Chitosan tripolyphosphate/TiO_2_	618.7	RO-16	46
S-doped lignin sulfonate carbons (LS-C12)	466	RO-16	This study

## Preliminary electrochemical measurements as supercapacitors

**Fig. 7:**
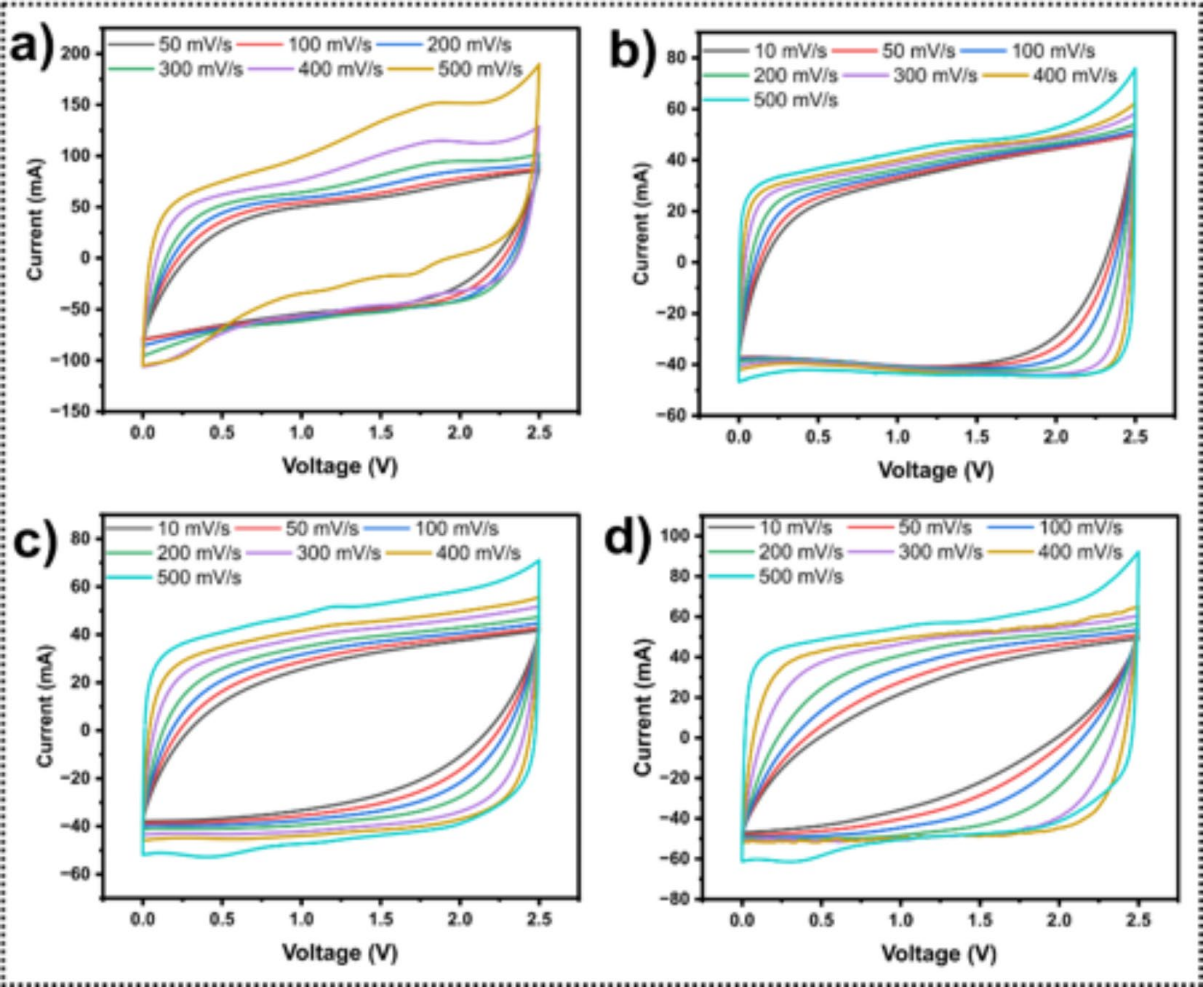
Cyclic voltammograms at different scan rates (a) LS-C3, (b) LS-C5, (c) LS-C6, and (d) LS-C8.

The voltammogram measurements give indispensable information about the current potential behavior and the charge storage reversibility mechanism of the electrode at scan rates of 10, 50, 100, 200, 300. 400, and 500 mV/s (Fig. 7a, b,c, d). The voltammogram of the chosen LS-C samples displays a semi-rectangular shape even at high scan rates, which are typical for carbonaceous materials with high specific surface areas, showing elevated capacitance values, basically due to the electric-double layer (EDLC) formation contribution. This result shows the good behavior of an EDLC capacitor with fast ion diffusion, good charge transfer, and potential change for all four samples. Furthermore, pseudo-capacitance contribution also is present in the LS-C electrodes [47], mainly due to the high concentration of sulfur atoms, as can be observed for sample LS-C8 – Fig. 7d with higher relative content of sulfur - Zn: S = 2:0.5. In this condition, the typical profile of the curve migrates from a square shaped to an oblate one with the increasing scan rate.

**Fig. 8:**
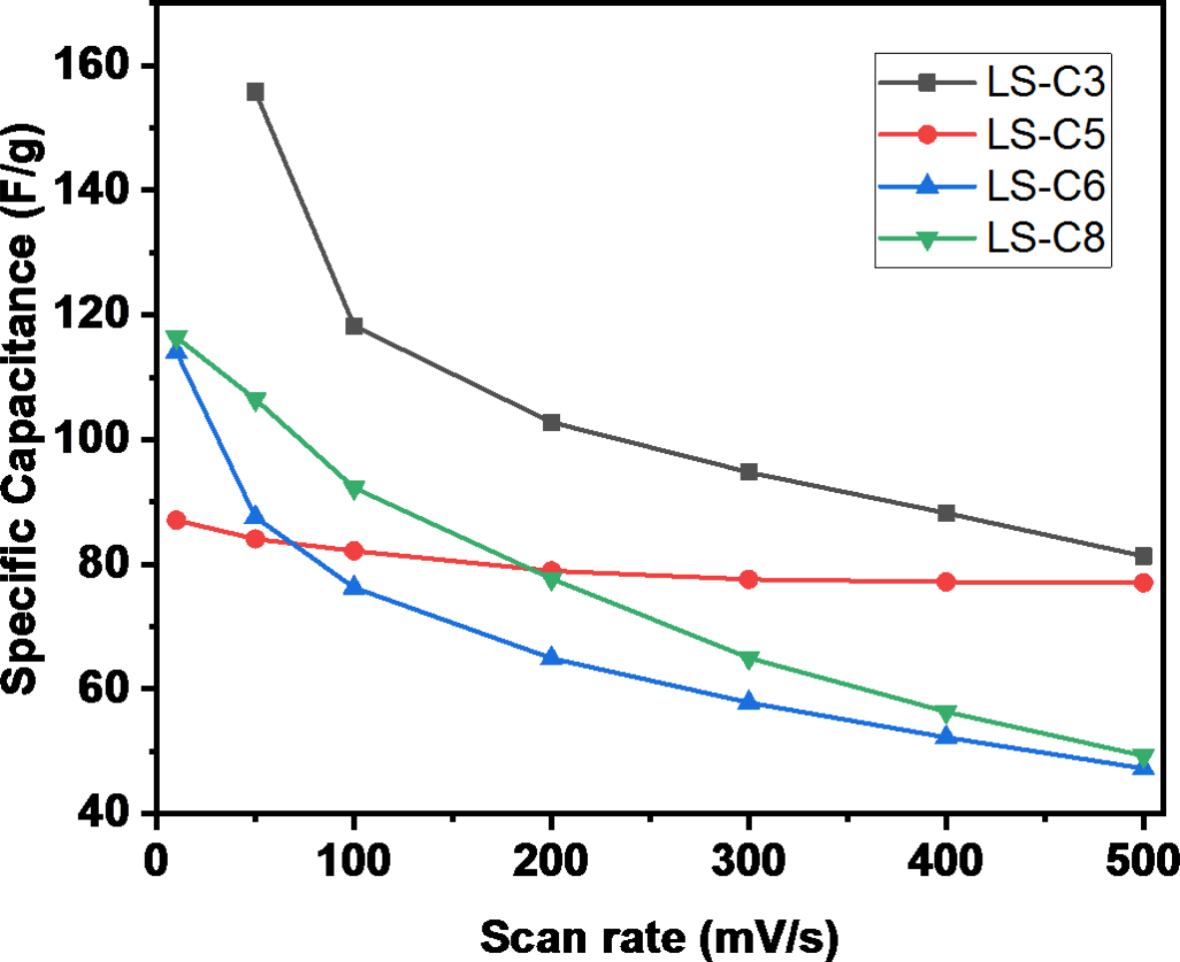
Specific capacitance as a function of scan rate for samples LS-C3, LS-C5, LS-C6, and LS-C8.

The CV technique can be conveniently explored for the calculus of the specific capacitance values from the following Eq.

1 $$\:{C}_{sp}=\frac{Area}{2*\upsilon\:*\varDelta\:V*mass}$$

where ν is the scan rate, ΔV is the voltage window, and mass is the total mass of the electrodes. The specific capacitance values obtained at the respective scan rates are given in Fig. 8. As expected, the capacitance is higher at lower scan rates and monotonically decreases at increasing scan rate. As can be observed, the order in performance for specific capacitance follows the order: LS-C3 > LS-C8 > LS-C6 > LS-C5. The best performance is mutually dependent on two important parameters: the S-doping improved the electrochemical performance of the LS-C doped electrode materials (0.5 and 0.25 for samples LS-C3 and LS-C8, respectively with zero for others) but also from the BET surface area of the electrodes: the best surface area for samples LS-C3 (1993 m^2^g^−1^) and for LS-C8 (1213 m^2^g^−1^) favoured the outstanding performance for these two experimental systems. The general decrease in the specific capacitance at increasing scan rates has been typically attributed to insufficient time for the electrolyte ions to complete the electrochemical reaction. This process is critical for samples with higher surface area that return a higher variation in the capacitance due to the required time to access active sites for charge accumulation (a reduced variation in the capacitance dependence with the scan rate is observed for samples with lower surface area – LS-C5).

**Fig. 9:**
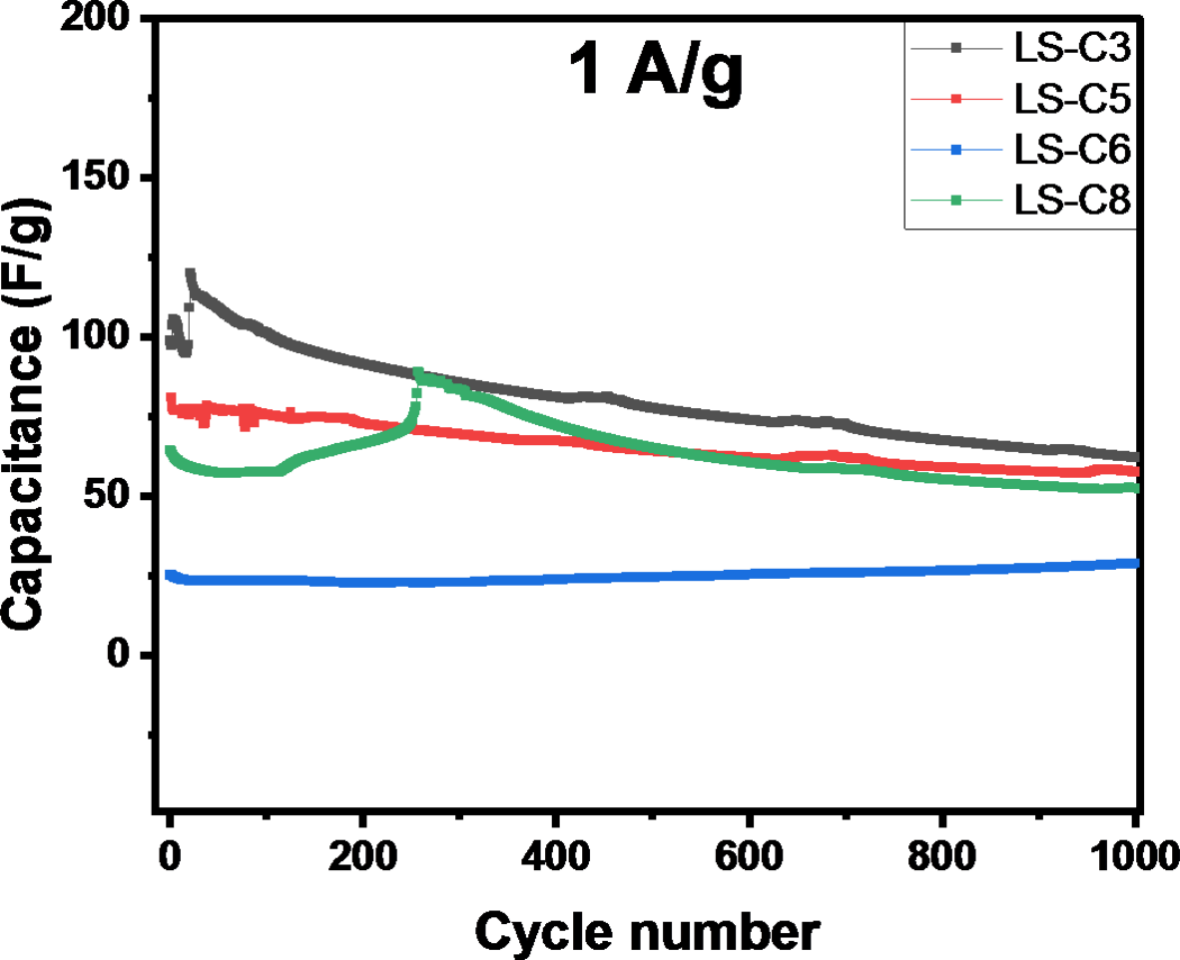
Electrochemical performance of LS-C3, LS-C5, LS-C6, and LS-C8 samples during the first 1000 cycles at a current of 1.0 A g^−1^.

Long-term cycling stability is an important aspect of supercapacitors’ performance. The standard evaluation of the device’s cyclability is performed from GCD curves at high current density (1Ag-1) by continuous cycling. Figure 9 shows the measured capacitance of devices as a function of the number of operation cycles.

As can be seen, and in agreement with results for specific capacitance vs. scan rate, minimal degradation is observed for samples with low surface area (samples LS-C5 and LS-C6). On the other hand, an anomalous event is observed for S-doped samples LS-C3 and LS-C8, which is observed as a first step with an increase in the capacitance for the following effective degradation of the material. This process has been reported in the literature^48^ and attributed to a self-activation process in which new pathways are created under repeated operation of the device, resulting in a transitory improvement of the capacitive performance of the device. This process is more evident for the S-doped samples with higher surface area and possible intricate structures for current circulation between electrolyte and electrodes.

Table 5compares the overall performance of LS-C-based devices with reported supercapacitors in the literature^49–56^, confirming the potential of doped material as an active electrode for supercapacitors, since the doped LS-C devices presented very good performances compared to the ones displayed in Table 5.

For instance, a supercapacitor device based on a carbon nanotubes/bi-metal composite (ZnMn_2_O_4_-MWCNTs) [50], exhibited a gravimetric capacitance of only 19.56 F/g and it is a much more complex and expensive material. The same logic applies to the supercapacitors fabricated with sulfur-doped carbon nanotubes that exhibited a capacitance of only 27 F/g.

Thus, we can safely state that due to the less complicate synthesis process of the LS-C materials and its respective supercapacitors performances, they can easily be considered promissing candidates for SCs electrode design with excellent performances.

**Table 5:** The capacitance of different electrode materials in comparison with present work.

Electrode material	Capacitance	Electrolyte	Current density (A g^−^1) or Scan rate (mV s^−1^)	Ref.
Graphene-ZnO composite	72 F/g	1 M KCl	100 mV s^−1^	49
ZnMn_2_O_4_-MWCNTs	19.56 F/g	1 M Na_2_SO_4_		50
N and S co-doped activated carbon	70 F/g	EmiFSI ionic liquid	1 A/g	51
ZnO@sulphur-doped carbon	38.36 F/g		100 mV s^−1^	52
Sulfur-doped carbon nanotubes	27 F/g	1 M TEABF_4_/acetonitrile	5 mA/cm	53
N and S co-doped nanoporous carbon	73 F/g	6 M KOH	1 A/g	54
KOH Activated CNTs	53.6 F/g	7 M KOH	1 A/g	55
N-doped MWNT	44.3 F/g	6 M KOH	1 mA/g	56
LS-C3	156 F/g	([BMIM]BF_4_	50 mVs^−1^	This work

## Conclusion

Waste biomass is a suitable raw material for materials synthesis with different potential applications. This work evaluated the potential application of lignin-sulfonate for synthesizing sulfur-doped carbon materials for water decontamination and supercapacitors applications. The preparation of the materials was optimised by employing statistical analysis and response surface methodology methods. The effect of pyrolysis temperature, ZnCl_2_/impregnation ratio, and sulfur content on the A_BET_ and A_MESO_were investigated. The as-prepared lignin-sulfonated materials (LS-Cs) exhibited very large surface areas despite of the preparation and doping conditions (475 to 1993 m^2^ g^−1^). The sulfur doping helped to introduce a large amount of sulfur atoms in the carbon materials up to 15.54 wt%. According to the statistical analysis, the activation temperature and sulfur-doping mainly affected the specific surface and mesopore areas. When tested as adsorbents, the LS-C materials displayed very large adsorption capacities for both tested molecules (197–372 mg g^[-1^) for DCF and (223–466 mg g^[-1^) for RO-16, larger than many reports found in the literature. Furthermore, when tested as electrodes in supercapacitors, the LS-C materials exhibited competitive capacitances even higher than many electrode materials from the literature.

This systematic study helps to pave the way to successfully design the synthesis of sulfur-doped carbons with improved adsorpvite and electrochemical performances. Besides, using lignin-sulfonate as a suitable resource to produce multifunctional materials provides a comprehensive solution to a proper and efficient waste management process and preparing sustainable materials for environmental and energy storage applications. However, deeper studies are needed to better understand how the sulfur doping deeply affected the nano-micro-structure of the carbons. For that, a more systematic approach involves addressing the limited information on basic principles of the sulfur mechanism and how it is connected to the changes/formation of porous and surface area, and surface chemistry and thus the ability to adsorb pollutants and store energy. Therefore, a deeper evaluation of designing the sulfur doping process together with the pyrolysis and activation methods to target both environmental and energy storage applications is needed. Furthermore, full electrochemical tests as supercapacitors will be conducted to fully evaluate the ability of the LS-C materials in supercapacitors.

## Data Availability

The data that support the findings of this study are available from the corresponding author upon reasonable request.
